# Who Tweets with Their Location? Understanding the Relationship between Demographic Characteristics and the Use of Geoservices and Geotagging on Twitter

**DOI:** 10.1371/journal.pone.0142209

**Published:** 2015-11-06

**Authors:** Luke Sloan, Jeffrey Morgan

**Affiliations:** 1 School of Social Sciences, Cardiff University, Cardiff, Wales; 2 School of Computer Science and Informatics, Cardiff University, Cardiff, Wales; University of Warwick, UNITED KINGDOM

## Abstract

In this paper we take advantage of recent developments in identifying the demographic characteristics of Twitter users to explore the demographic differences between those who do and do not enable location services and those who do and do not geotag their tweets. We discuss the collation and processing of two datasets—one focusing on enabling geoservices and the other on tweet geotagging. We then investigate how opting in to either of these behaviours is associated with gender, age, class, the language in which tweets are written and the language in which users interact with the Twitter user interface. We find statistically significant differences for both behaviours for all demographic characteristics, although the magnitude of association differs substantially by factor. We conclude that there are significant demographic variations between those who opt in to geoservices and those who geotag their tweets. Not withstanding the limitations of the data, we suggest that Twitter users who publish geographical information are not representative of the wider Twitter population.

## Introduction

The advent of the ‘Big Data’ revolution presents both great opportunities and great challenges to 21^st^ Century Social Science. In 2013 the UK Department of Business, Innovation and Skills calculated that the previous two years had seen the generation of 90% of the world’s data through the exponential increase in digital activity [1]. Whereas social scientists used to hold the monopoly on data relating to human beliefs, values, attitudes and behaviour, this is no longer the case and the slow reaction of the social sciences to respond and embrace these new forms of data has been lamented [2]. In response to this, recent work has focused on how this ‘naturally occurring data’ can be conceptually integrated into the social sciences [3] and interdisciplinary work has looked at how collective behaviour in the real world can be understood through big data [4][5][6], how computer science and criminology can come together to map and understand cyberhate [7][8] and how Twitter can be used to predict (or offer post-event analysis of) elections [9][10][11], although not without some serious methodological criticisms [12][13]. Twitter use on mobile phones has even been used as a proxy to estimate crowd sizes [14]

Indeed, such methodological criticisms arise precisely because of the new nature of the data and the fact that methodological investigations are still in their infancy. In the case of Twitter, although such data is easily accessible and has the potential to inform us on how people feel, what they believe and how they react to real world events in real time, it lacks the demographic information which allows social scientists to make group comparisons [15]. Much work has been conducted to address this deficit through the development of proxy demographics for Twitter users around characteristics such as location, gender, language, age and social class [16][17][18][19]. This work has demonstrated that the population of Twitter users in the UK differs significantly from the wider UK population in the sense that users are younger and there appears to be a disproportionately high number of users from lower managerial, administrative and professional occupations (NS-SEC 2) alongside an under-representation of users in lower supervisory, semi-routine and routine occupations (NS-SEC 5, 6 and 7) [17], but the distribution between male and female users (for those where gender can be identified) is the same amongst UK Twitter users as in the UK 2011 Census [16].

This paper builds upon this methodological body of work by cross-referencing demographic proxies to understand the differences between those who enable geoservices and geotag their tweets, and those who do not.

## Why Location Matters

Twitter users have the option to enable location services on their account. This feature is off by default and requires users to opt in, but once it is enabled users can geotag their tweets with precise location data in the form of latitude and longitude [20]. Previous studies demonstrate that approximately 0.85% of tweets are geotagged, meaning that the exact position of where the tweeter was when the tweet was posted is recorded using longitude and latitude measurements [16].

Only tweets with original content can be geotagged. Retweets generated by invoking the retweet command in the Twitter user interface are not classed by Twitter as original content and are never geotagged. However, retweets generated by copying and pasting the content of a tweet into the tweet-composition box are classed as original content and can be geocoded (if the user chooses).

From a social scientific perspective this location data is incredibly valuable as it enables us to establish the geographic context in which the tweeter is immersed at the point of data creation. Having a geo-spatial point enables us to position tweets within existing geographies to which demographic and contextual data can be linked, thus overcoming criticisms of social media sources being ‘data-light’ [13]. For example, tweets about fear or insecurity could be located within neighbourhoods for which crime data is recorded, potentially illustrating whether there is a link between actual recorded deviant behaviour and perceptions of safety. On the run up to an election, tweets in favour of particular candidates and/or parties can be located within parliamentary constituencies to offer a picture of which way the electorate might vote—although this is actually methodologically challenging [9].

For tweets that are not geotagged we can begin to identify proxy geographical measures [16], but the area that users refer to in their public profiles are typically too broad to be of use. Knowing that someone is from ‘Cardiff’, ‘Manchester’ or even ‘East London’ does not allow us to locate them within established official geographies. Larger areas also mean higher levels of demographic heterogeneity, making it hard to classify what is different about an area e.g. are it useful to talk about the effect of demographic context on tweets in Cardiff and Manchester?

There is also a conceptual difference between geo-tagging and profile-based locations. Geotagged data tell us where a person is when they publish the tweet, whilst profile data could tell us any number of things including where people were born, lived, employed, are passing through or simply identify with. For all these reasons, geotagged tweets have become the gold standard. They contain the most information in the most useful and accurate format.

Although the proportion of geotagged tweets seems small, this actually accounts for over 4 million tweets every 24 hours using an estimate of 500 million tweets per day [21]. Given that one of the problems with ‘big data’ is associated with the storage and processing demands of managing velocity and volume [22], the logical step for most social scientific analysis is to optimise data collection strategies by collecting subsamples of data. Clearly these subsamples should contain the most valuable information, thus it may be considered rational to target data collection towards geotagged tweets only.

The effectiveness of this strategy is certainly dependent on the research question being addressed. For investigations into larger geographies at city, regional or national level a researcher may choose to cast their data collection net more broadly. It is certainly the case that the small proportion of tweets that are geotagged can have adverse effects on sampling, particularly if one is collecting all tweets containing specific terms [9] and the geo-coding condition in turn only returns 0.85% of the potential pool of eligible data. However for researchers interested in the intersection between Twitter and other forms of traditional administrative data (i.e. the augmentation thesis [3]), geotagged tweets are the obvious source to target.

Having made a case for the primacy of this special 0.85% of Twitter traffic, there is significant concern over who has enabled location services on their account. Fundamentally this is a question about representativeness, not in relation to the Twitter population as a subset of the general population but whether this group is representative of other Twitter users. Do those who have location services enabled constitute a random sample of the Twitter population or are they significantly different? Graham et al. [23] discuss this issue and suggest that “it is unlikely that they form a representative sample of the broader universe of content (i.e., the division between geotagged and non-geotagged users is almost certainly biased by factors such as socioeconomic status, location, and education)” however this is only a hypothesis–and one that is yet to be tested.

So far no work has been done on analysing the demographic differences between those with geo-tagging and those without because social media data, particularly that ascertained from Twitter, is often lacking in demographic information [15]. However recent work on the development of demographic proxies as part of the COSMOS program of work [24][25] has resulted in tools for estimating a range of demographic characteristics including: language and gender [16]; age for all countries and occupation with social class (NS-SEC) for UK users [17]. Records harvested from the Twitter API also include metadata fields for each user and tweet including the time zone specified by user, the Twitter user-interface language and whether location services are enabled.

Following these developments the aim of this paper is fundamentally quite simple–using a dataset of individual Twitter users we investigate whether there are any significant differences in the demographic and profile characteristics of users with and without geographical data treating the 1% feed as the population. We distinguish between users who have location services enabled and those who actually geotag their tweets within the study timeframe. We present separate analyses for these two groups because (as we demonstrate) there is a notable disparity between the proportions of those who enable the global setting and those who actually attach geodata to individual tweets.

The two research questions can be framed thus:

RQ1: Are there any demographic differences between users who do or do not enable local services on Twitter?

RQ2: Are there any demographic differences between users who do or do not geotag their tweets?

The first question is concerned with the preferences of a user and their general attitude towards using locations services. As an example, if we find that users in certain locations are more likely to enable this setting than others then we might expect this disparity to manifest in actual geotagged tweets. Enabling the global setting is a necessary but not sufficient condition of geotagging as users can choose not to geotag tweets on a case-by-case basis.

The second question addresses the representativeness of users who commit to geotagging individual tweets compared to those who don’t. Critically, by using individual tweet measures the ‘*those who don’t*’ group can include users who have the global setting enabled but do not actually allow their location to be associated with their tweets. If there are no discernible differences on the range of measures being tested then users who geotag their tweets can reasonably be considered as representative of the wider Twitter population (defined here as the 1% feed) and, because the 1% feed is defined as random, can thus be used in the same way as any probability sample for a social survey assuming that all Twitter users are the population of interest. Alternatively if there are differences between the two groups then we will know what they are, enabling researchers to consider methods for ameliorating or controlling for such discrepancies or simply account for the limitations of the data.

## Data

For this analysis it was necessary to construct two datasets–one for investigating location services and another for geotagged tweets. All data was collected using the free 1% feed of the Twitter API during April 2015. Whenever a user tweeted during this period, their profile data was collected and stored. For the location services dataset (‘Dataset1’) we simply used the profile data associated with a user’s most recent tweet, resulting in a dataset of 30,020,446 unique tweeters.

The specification for the dataset on whether users use geotagging on tweets or not (‘Dataset2’) is more complex because the dynamic behaviour of users in relation to geotagging means that simply taking the last tweet may not be appropriate. Therefore, whenever a user tweeted during this period, their profile data was collected and stored. We then looked at all the tweets associated with their account to see if any were geotagged and took the profile data that was accurate when this tweet was posted–this is one way in which to derive a single metric from multiple records. The resulting dataset is a list of users with a binary flag for whether any tweets collected during the study period were geotagged or not. For users with no geotagged tweets we simply take their latest tweet as the reference point for sourcing their profile information, however these users may still have location services enabled.

For some users, all of the records we have may be retweets (which cannot be geotagged) and this needs to be dealt with differently for each research question. For RQ1 we do not exclude retweets because we are interested in the global settings of users (‘Dataset1’). For RQ2 we do exclude retweets because we are interested in the decisions that users make when they post a tweet *that could be geotagged* (‘Dataset2’). This means that the dataset for RQ2 is substantially reduced to 23,789,264 cases and that we picked up only retweets for 6,231,182 or 20.8% of users during the study period.

Although there is some work that questions whether the 1% API is random in relation to tweet context such as hashtags and LDA analysis [26], Twitter maintains that the sampling algorithm is “completely agnostic to any substantive metadata” and is thus “a fair and proportional representation across all cross-sections” [27]. Because we would not expect any systematic bias to be present in the data due to the nature of the 1% API stream we consider this data to be a random sample of the Twitter population. We also have no *a priori* reason for thinking that users tweeting in April 2015 are not representative of the population and we can therefore apply inferential statistics and significance tests to evaluate hypotheses concerning whether any differences between those with geoservices and geotagging enabled differ to those who don’t. There may well be users who have generated geotagged tweets who are not picked up in the 1% API stream and this will always be a limitation of any research that doesn’t use 100% of the data and is an important qualification in any research using this data source.

Twitter terms and conditions prevent us from openly sharing the metadata supplied by the API, therefore ‘Dataset1’ and ‘Dataset2’ contain only the user ID (which is acceptable) and the demographics we have derived: tweet language, gender, age and NS-SEC. Replication of this study can be conducted through individual researchers using user IDs to gather the Twitter-produced metadata that we cannot share.

## Analysis

### Location Services vs. Geotagging Individual Tweets

Looking at all users (‘Dataset1’), overall 58.4% (n = 17,539,891) of users do not have location services enabled whilst 41.6% do (n = 12,480,555), thus indicating that most users do not choose this setting. Having said that, the proportion of those with the setting enabled is high considering that users have to opt in. When excluding retweets (‘Dataset2’) we see that 96.9% (n = 23,058166) have no geotagged tweets in the dataset whilst 3.1% (n = 731,098) do. This is much higher than previous estimates of geotagged content of around 0.85% [16] because the focus of this study is on the proportion of *users* with this characteristic rather than the proportion of *tweets*. However, it is notable that although a substantial proportion of users enabled the global setting, very few then go on to actually geotag their tweets–thus demonstrating clearly that enabling locations services is a necessary but not sufficient condition of geotagging.

### Gender

Table 1 is a crosstabulation of whether location services are enabled and gender (identified using the method proposed by Sloan et al. 2013 [16]). Gender could be identified for 11,537,140 individuals (38.4%) and there is a slight preference for males to be less likely to enable the setting than females or users with names classified as unisex. There is a clear discrepancy in the unknown group with a disproportionate number of users opting for ‘not enabled’ and as the gender detection algorithm looks for an identifiable first name using a database of over 40,000 names, we may observe that there is an association between users who do not give their first name and do not opt in to location services (such as organisational and business accounts or those conscious of maintaining a level of privacy). When removing the unknowns the relationship between gender and enabling location services is statistically significant (x^2^ = 117324.18, 3 df, p<0.001) as is the effect size despite being very small (Cramer’s V = 0.008, p<0.001).

**Table 1:** Crosstabulating whether location services are enabled and gender (‘Dataset1’).

Gender:	Not Enabled:	Enabled:	Total:
Male	55.0% (n = 2,864,158)	45.0% (n = 2,346,886)	5,211,044
Female	54.2% (n = 2,776,220)	45.8% (n = 2,345,110)	5,121,330
Unisex	54.1% (n = 651,809)	45.9% (n = 552,957)	1,204,766
Unknown	60.9% (n = 11,247,704)	39.1% (n = 7,235,602)	18,483,306
**Total:**	58.4% (n = 17,539,891)	41.6% (n = 12,480,555)	30,020,446

Table 2 presents the relationship between gender and whether a user produced a geotagged tweet during the study period. Male users are more likely to geotag their tweets then female users, but only by an increase of 0.1%. Users for which the gender is unknown show a lower geotagging rate, but most interesting is the gap between unisex geotaggers and male/female users, which is notably larger for geotagging than for enabling location services. This means that although similar proportions of users with unisex names enabled location services as those with male or female names, they are notably less likely to geotag their tweets than male or female users. When removing unknowns the difference is statistically significant (x^2^ = 1114.602, 2 df, p<0.001) with a small effect size (Cramer’s V = 0.011, p<0.001).

**Table 2:** Crosstabulating geotagging users and gender (‘Dataset2’).

Gender:	Not Used Geotagging:	Used Geotagging:	Total:
Male	95.8% (n = 4,031,850)	4.2% (n = 175,265)	4,207,115
Female	95.9% (n = 4,088,213)	4.1% (n = 174,922)	4,263,135
Unisex	96.5% (n = 1,018,140)	3.5% (n = 36,496)	1,054,636
Unknown	97.6% (n = 13,919,963)	2.4% (n = 344,415)	14,264,378
**Total:**	96.9% (n = 23,058,166)	3.1% (n = 731,098)	23,789,264

### Age

Detecting the age of users on Twitter is not without its problems (see Sloan et al. for extensive discussion [17]) and the analysis that follows should be treated carefully as misclassifications due to humour and deceit are unavoidable. To limit extreme instances of this, the age detection algorithm ignores ages below 13 years (the legal age for using Twitter) and above 100 years. Of the 30,020,446 cases in ‘Dataset1’, age could be derived for 54,484 (0.18%) of users. This is lower than the 0.37% of users successfully categorised by previous studies [17] but accounts for the fact that this dataset includes non-English language profiles which the detection tool cannot process.

**Fig 1:**
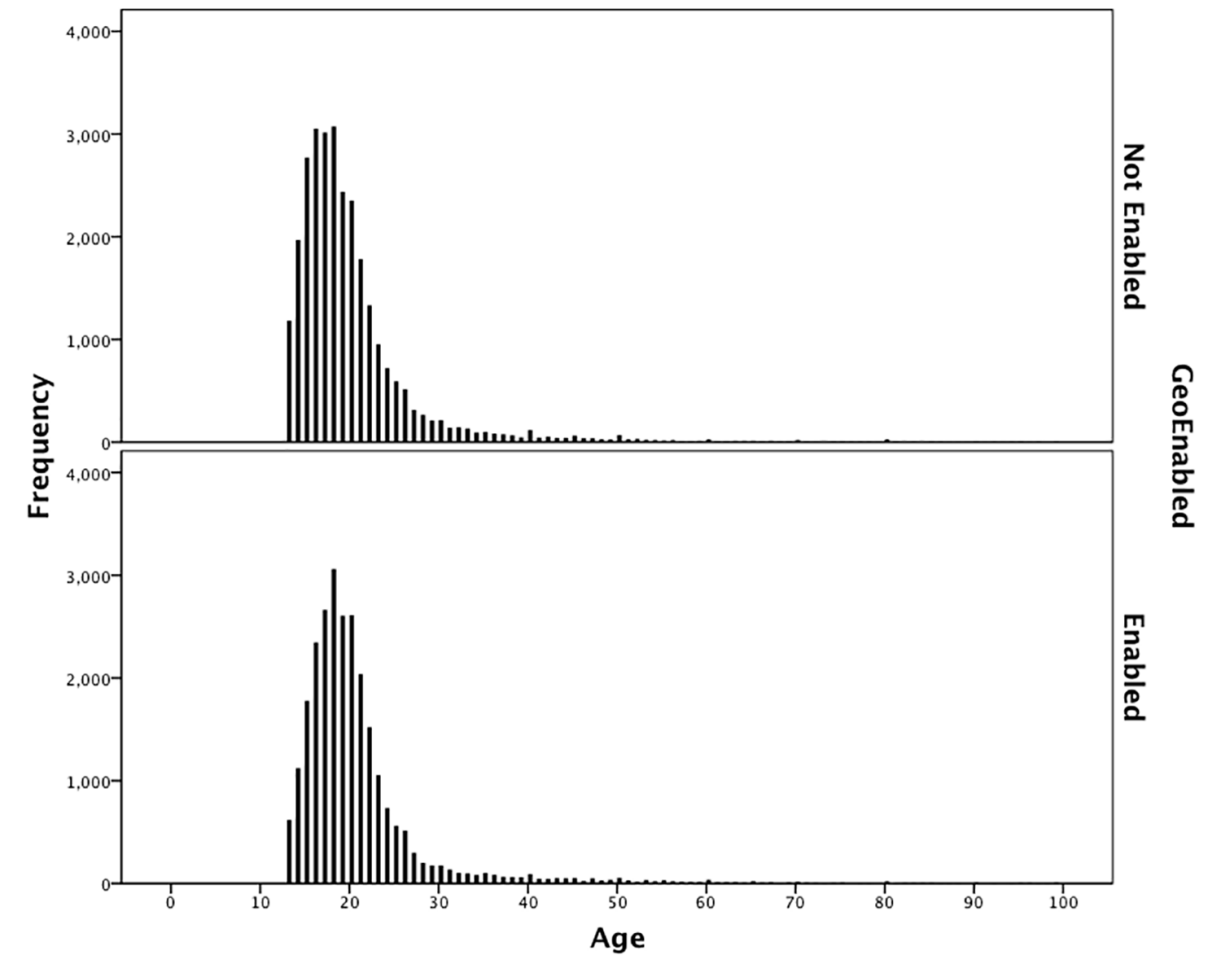
Comparing the age distribution of users who do and do not enable location services.

Fig 1 illustrates the two distributions of age for those who do enable location services and those who do not. There is a long tale on both, but notably the tail has a less steep decline on the right-hand side for those without the setting enabled. An independent samples Mann-Whitney U confirms that the difference is statistically significant (p<0.001) and descriptive measures show that the mean age for ‘not enabled’ is lower than for ‘enabled’ at 20.30 and 20.84 respectively and higher medians (18.00 and 19.00 respectively) with a slightly higher standard deviation for ‘not enabled’ (8.44) than ‘enabled’ (8.171). This indicates an association between older users and opting in to location services. One explanation for this might be a naivety on the part of older users over enabling location based services, but this does assume that younger users who are more ‘tech savvy’ are more reticent towards allowing location based data.

**Fig 2:**
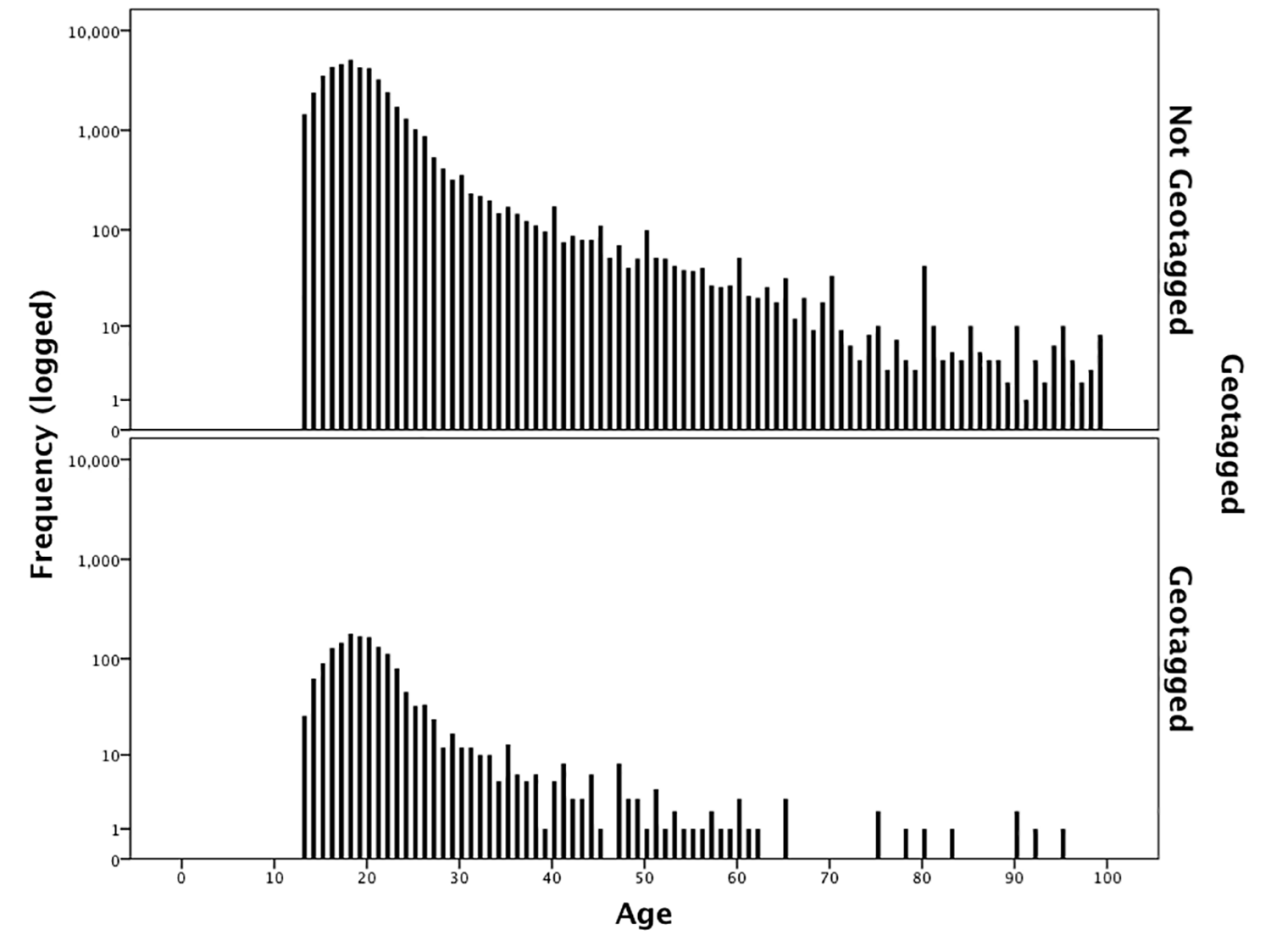
Comparing the age distribution of users who do and do not geotag tweets (*y*-axis logged).

Fig 2 shows the distribution of age for users who produced or did not produce geotagged content (‘Dataset2’). Of the 23,789,264 cases in the dataset, age could be identified for 46,843 (0.2%) users. Because the proportion of users with geotagged content is so small the *y-axis* has been logged. There is a statistically significant difference in the age profile of the two groups according to an independent samples Mann-Whitney U test (p<0.001) with a mean age of 20.76 for non-geotaggers and 21.58 for geotaggers (medians of 19.00 and 20.00 respectively), indicating that there is a tendency for geotaggers to be slightly older than non-geotaggers.

### Class (NS-SEC)

Following on from recent work on classifying the social class of tweeters from profile meta-data (operationalised in this context as NS-SEC–see Sloan et al. for the full methodology [17]), we apply a class detection algorithm to our data to investigate whether certain NS-SEC groups are more or less likely to enable location services. Although the class detection tool is not perfect, previous studies have shown it to be accurate in classifying certain groups, notably professionals [17]. General misclassifications are associated with occupational terms with other meanings (such as ‘page’ or ‘medium’) and jobs that may also be termed hobbies (such as ‘photographer’ or ‘painter’). The possibility of misclassification is an important limitation to consider when interpreting the results, but the important point is that we have no *a priori* reason for believing that misclassifications would not be randomly distributed across those with and without location services enabled. With this in mind, we are not so much interested in the overall representation of NS-SEC groups in the data as the *proportional differences* between location enabled and non-enabled tweeters.

NS-SEC can be harmonised with other European measures, but the occupation detection tool is designed to pick-up UK occupations only and it should not be applied outside of this context. Previous studies have identified UK users using geotagged tweets and bounding boxes [16][17], but since the purpose of this paper is to compare this group with other non-geotagging users we decided to use time zone as a proxy for location. The Twitter API provides a time zone field for each user and the following analysis is limited to users associated with one of the two GMT zones in the UK: Edinburgh (n = 28,046) and London (n = 597,197).

Table 3 presents the relationship between NS-SEC and location services. There is a statistically significant association between the two variables (x^2^ = 54.489, 6 df, p<0.001) but the effect is weak (Cramer’s V = 0.028, p<0.001). There is only a difference of 4.6% between the lowest and highest rates of enabling geoservices across NS-SEC groups with the tweeters from semi-routine occupations the most likely to allow the setting. Why those in routine occupations should have the lowest proportion of enabled users is unclear, but the size of the difference is enough to demonstrate that the categorisation tool is measuring a demographic characteristic that does seem to be associated with differing patterns of behaviour.

**Table 3:** Crosstabulating NS-SEC with whether location services are enabled.

NS-SEC	Label	Not Enabled	Enabled	Total
1	Higher managerial, administrative & professional occupations	46.6% (n = 4299)	53.4% (n = 4931)	9230
2	Lower managerial, administrative & professional occupations	47.2% (n = 12685)	52.8% (n = 14171)	26856
3	Intermediate occupations	47.5% (n = 4153)	52.5% (n = 4593)	8746
4	Small employers & own account workers	45.1% (n = 2477)	54.9% (n = 3021)	5498
5	Lower supervisory & technical occupations	44.9% (n = 534)	55.1% (n = 654)	1188
6	Semi-routine occupations	43.6% (n = 3987)	56.4% (n = 5159)	9146
7	Routine occupations	48.2% (n = 3451)	51.8% (n = 3705)	7156
**Total:**		46.6% (n = 31586)	53.3% (n = 36234)	67820

Table 4 explores the association between NS-SEC and whether a user geotags or not. The relationship is statistically significant (x^2^ = 16.182, 6 df, p = 0.013) but the effect is even weaker than for enabling location services (Cramer’s V = 0.016, p = 0.013) with a difference of only 0.9% between the most and least likely groups to geotag. Interestingly, small employers and own account workers have the same level of geotagging as semi-routine occupations (4.2%) whilst the former group has a lower proportion of users with location services enabled. As the decrease in those who geotag is not standard across all groups we can observe that the mechanisms and processes that link enabling geoservices and actually geotagging a tweet are inflected to different degrees by NS-SEC group.

**Table 4:** Crosstabulating NS-SEC with geotagging users.

NS-SEC	Label	Not Geotagged	Geotagged	Total
1	Higher managerial, administrative & professional occupations	96.1% (n = 7,618)	3.9% (n = 308)	7,926
2	Lower managerial, administrative & professional occupations	96.5% (n = 22,935)	3.5% (n = 835)	23,770
3	Intermediate occupations	96.4% (n = 7,359)	3.6% (n = 274)	7,633
4	Small employers & own account workers	95.8% (n = 4,873)	4.2% (n = 215)	5,088
5	Lower supervisory & technical occupations	95.9% (n = 984)	4.1% (n = 42)	1,026
6	Semi-routine occupations	95.8% (n = 7,658)	4.2% (n = 335)	7,993
7	Routine occupations	96.7% (n = 6,152)	3.3% (n = 209)	6,361
**Total:**		96.3% (n = 57,579)	3.7% (n = 2,218)	59,797

### User Interface Language

The language of the Twitter user interface is the language that the user chooses to interact with and not necessarily the language that they choose to tweet in. When comparing user interface language with whether location service are enabled or not we find 123 different languages, many of which are in single of double figures, therefore we present only the 20 most frequently occurring user interface choices in Table 5 below. There is a statistically significant association between user interface language and whether location services are enabled both when taking only the top 20 (x^2^ = 834802.60, 122df, p<0.001) and all languages (x^2^ = 826471.01, 19df, p<0.001) although the latter is undermined by 48.8% of cells having an expected count of less than 5, hence the need to be selective.

**Table 5:** Crosstabulating Twitter user interface language (top 20) with whether location services are enabled.

Language:	Code:	Not Enabled:	Enabled:	Total:
English	en	56.6% (n = 8143700)	43.4% (n = 6234416)	14378116
Japanese	ja	69.4% (n = 3603473)	30.6% (n = 1589842)	5193315
Spanish	es	48.8% (n = 1764145)	51.2% (n = 1848193)	3612338
Arabic	ar	70.1% (n = 919952)	29.9% (n = 391723)	1311675
Portuguese	pt	43.0% (n = 516463)	57.0% (n = 685814)	1202277
Turkish	tr	52.1% (n = 468241)	47.9% (n = 431067)	899308
Russian	ru	77.2% (n = 573039)	22.8% (n = 169713)	742752
French	fr	57.1% (n = 405485)	42.9% (n = 304086)	709571
Indonesian	id	44.4% (n = 273780)	55.6% (n = 342348)	616128
Korean	ko	73.2% (n = 202514)	26.8% (n = 74000)	276514
Italian	it	56.1% (n = 135032)	43.9% (n = 105877)	240909
Thai	th	55.5% (n = 124466)	44.5% (n = 99627)	224093
German	de	72.5% (n = 139954)	27.5% (n = 53126)	193080
Dutch	nl	61.3% (n = 74685)	38.7% (n = 47184)	121869
Swedish	sv	60.2% (n = 29872)	39.8% (n = 19711)	49583
Chinese	zh	75.2% (n = 35025)	24.8% (n = 11558)	46583
Polish	pl	62.6% (n = 27172)	37.4% (n = 16208)	43380
Finnish	fi	63.8% (n = 15840)	36.2% (n = 8989)	24829
Catalan	ca	53.4% (n = 11723)	46.6% (n = 10250)	21973
Greek	el	67.4% (n = 12320)	32.6% (n = 5958)	18278
**Total:**		58.4% (n = 17476881)	41.6% (n = 12449690)	29926571

Table 5 shows clear differences with Russian-language user interface users being the least likely to enable location settings (22.8%), closely followed by those who interact in Chinese (24.8%), Korean (26.8%) and German (27.5%). Those most likely to enable the settings use the Portuguese interface (57.0%) followed closely by Indonesian (55.6%), Spanish (51.2%) and Turkish (47.9%). One may speculate as to why these differences occur in relation to cultural and political contexts, but the differences in preference are clear and obvious.

The same analysis of the top 20 countries for users who do and do not geotag shows the same top 20 countries (Table 6) and, as above, there is a significant association between the behaviour and language of interface (x^2^ = 235285.01, 19df, p<0.001). However, although Russian-language user interface users were the least likely to enable location settings they by no means have the lowest geotagging rate (2.5%). It is Korean interface users that are the least likely to actually geotag their content (0.3%) followed closely by Japanese (0.8%), Arabic (0.9%) and German (1.3%). Those who use the Turkish interface are the most likely to use geotagging (8.8%) then Indonesian (6.3%), Portuguese (5.7%) and Thai (5.2%).

**Table 6:** Crosstabulating interface language (top 20) with whether users geotag or not.

Language:	Code:	Not Geotagged	Geotagged	Total:
English	en	96.6% (n = 10719718)	3.4% (n = 373678)	11093396
Japanese	ja	99.2% (n = 4673294)	0.8% (n = 37175)	4710469
Spanish	es	95.7% (n = 2583704)	4.3% (n = 115959)	2699663
Portuguese	pt	94.3% (n = 958167)	5.7% (n = 57611)	1015778
Arabic	ar	99.1% (n = 990961)	0.9% (n = 8762)	999723
Russian	ru	97.5% (n = 637263)	2.5% (n = 16632)	653895
Turkish	tr	91.2% (n = 574730)	8.8% (n = 55530)	630260
French	fr	97.4% (n = 516445)	2.6% (n = 13781)	530226
Indonesian	id	93.7% (n = 433745)	6.3% (n = 29310)	463055
Korean	ko	99.7% (n = 226643)	0.3% (n = 644)	227287
Italian	it	96.9% (n = 181098)	3.1% (n = 5848)	186946
German	de	98.7% (n = 147140)	1.3% (n = 1978)	149118
Thai	th	94.8% (n = 108667)	5.2% (n = 5910)	114577
Dutch	nl	97.1% (n = 90554)	2.9% (n = 2690)	93244
Chinese	zh	97.9% (n = 35956)	2.1% (n = 786)	36742
Polish	pl	98.6% (n = 35372)	1.4% (n = 505)	35877
Swedish	sv	96.7% (n = 32924)	3.3% (n = 1137)	34061
Catalan	ca	96.4% (n = 13177)	3.6% (n = 498)	13675
Greek	el	97.9% (n = 11622)	2.1% (n = 245)	11867
Finnish	fi	98.0% (n = 11035)	2.0% (n = 222)	11257
**Total**		96.9% (n = 22982215)	3.1%(n = 728901)	23711116

Besides speculation over why these differences occur, Tables 5 and 6 demonstrate that there is a user interface language effect in play that shapes behaviour both in whether location services are enabled and whether a user uses geotagging. User interface language is not a proxy for location so these cannot be dubbed as country level effects, but perhaps there are cultural differences in attitudes towards Twitter use and privacy for which interface language acts as a proxy.

### User Tweet Language

The language of individual tweets can be derived using the Language Detection Library for Java [16]. 66 languages were identified in the dataset and the language of the last tweet of 1,681,075 users could not be identified (5.6%). There is a statistically significant association between these 67 languages and whether location services are enabled (x^2^ = 1050644.2, 65df, p<0.001) but, as with user interface language, we present the 20 most frequently occurring languages below in Table 7 (x^2^ = 1041865.3, 19df, p<0.001).

**Table 7:** Crosstabulating tweet language (top 20) with whether location services are enabled.

Language:	Code:	Not Enabled	Enabled	Total:
English	en	55.9% (n = 6232464)	44.1% (n = 4921668)	11154132
Japanese	ja	69.8% (n = 3699020)	30.2% (n = 1600458)	5299478
Spanish	es	49.1% (n = 1621107)	50.9% (n = 1679340)	3300447
Arabic	ar	70.5% (n = 1365579)	29.5% (n = 572310)	1937889
Portuguese	pt	41.5% (n = 481955)	58.5% (n = 678749)	1160704
Indonesian	in	44.2% (n = 499068)	55.8% (n = 629732)	1128800
Turkish	tr	54.0% (n = 489846)	46.0% (n = 418055)	907901
Russian	ru	81.8% (n = 676610)	18.2% (n = 150450)	827060
French	fr	56.2% (n = 327940)	43.8% (n = 256048)	583988
Korean	ko	71.1% (n = 243286)	28.9% (n = 99087)	342373
Thai	th	48.2% (n = 161163)	51.8% (n = 173226)	334389
Tagalog	tl	45.8% (n = 143638)	54.2% (n = 169922)	313560
Italian	it	54.4% (n = 110504)	45.6% (n = 92461)	202965
German	de	69.0% (n = 95398)	31.0% (n = 42916)	138314
Dutch	nl	58.1% (n = 72855)	41.9% (n = 52541)	125396
Swedish	sv	52.5% (n = 26326)	47.5% (n = 23851)	50177
Polish	pl	56.8% (n = 26953)	43.2% (n = 20487)	47440
Haitian	ht	53.6% (n = 24879)	46.4% (n = 21502)	46381
Estonian	et	55.8% (n = 24478)	44.2% (n = 19370)	43848
Ukrainian	uk	77.6% (n = 23306)	22.4% (n = 6716)	30022
**Total**		58.4% (n = 16346375)	41.6% (n = 11628889)	27975264

As when looking at interface language, users who tweeted in Russian were the least likely to have location services enabled (18.2%) followed by Ukrainian (22.4%), Korean (28.9%) and Arabic (29.5%) tweeters. Users writing in Portuguese were the most likely to have location services enabled (58.5%) closely trailed by Indonesian (55.8%), the Austronesian language of Tagalog (the official name for Filipino—54.2%) and Thai (51.8%).

We present a similar analysis of the top 20 languages for in Table 8 (using ‘Dataset2’) for users who did and did not use geotagging. Note that the 19 of the top 20 most frequent languages are the same as in Table 7 with Ukrainian being replaced at 20^th^ position by Slovenian. The tweet language could not be identified for 1,503,269 users (6.3%) and the association is significant when only including the top 20 most frequent languages (x^2^ = 264821.40, 19df, p<0.001). As with user interface language in Table 6, the least likely groups to use geotagging are those who tweet in Korean (0.4%), followed by Japanese (0.8%), Arabic (0.9%), Russian and German (both 2.0%). Again, mirroring the results in Table 6, Turkish tweeters are the most likely to geotag (8.3%), then Indonesian (7.0%), Portuguese (5.9%) and Thai (5.6%).

**Table 8:** Crosstabulating tweet language (top 20) with users used geotagging or not.

Language:	Code:	Not Geotagged:	Geotagged:	Total:
English	en	96.7% (n = 8083523)	3.3% (n = 275321)	8358844
Japanese	ja	99.2% (n = 4762822)	0.8% (n = 38231)	4801053
Spanish	es	95.6% (n = 2343493)	4.4% (n = 107758)	2451251
Arabic	ar	99.1% (n = 1352119)	0.9% (n = 12707)	1364826
Portuguese	pt	94.1% (n = 931212)	5.9% (n = 58225)	989437
Indonesian	in	93.0% (n = 812496)	7.0% (n = 61579)	874075
Russian	ru	98.0% (n = 719338)	2.0% (n = 14816)	734154
Turkish	tr	91.7% (n = 567254)	8.3% (n = 51249)	618503
French	fr	97.1% (n = 427340)	2.9% (n = 12901)	440241
Tagalog	tl	95.7% (n = 298373)	4.3% (n = 13415)	311788
Korean	ko	99.6% (n = 256037)	0.4% (n = 1117)	257154
Thai	th	94.4% (n = 184890)	5.6% (n = 10899)	195789
Italian	it	96.3% (n = 159880)	3.7% (n = 6074)	165954
German	de	98.0% (n = 114658)	2.0% (n = 2331)	116989
Dutch	nl	96.6% (n = 98897)	3.4% (n = 3484)	102381
Polish	pl	97.8% (n = 42613)	2.2% (n = 978)	43591
Swedish	sv	95.5% (n = 40047)	4.5% (n = 1887)	41934
Haitian	ht	96.1% (n = 39975)	3.9% (n = 1621)	41596
Estonian	et	94.5% (N = 36023)	5.5% (n = 2094)	38117
Slovenian	sl	96.3% (n = 26653)	3.7% (n = 1031)	27684
**Total**		96.9% (n = 21297643)	3.1% (n = 677718)	21975361

It is possible that users tweet in multiple languages. The methodological decision to focus on the most recent tweet was made to enable a snapshot of Twitter users much akin to a cross-sectional social survey and this means that multiple language use is not accounted for. However we would not anticipate any systematic over-representation of a particular language used in most recent tweets due to the random nature of the 1% Twitter API and the fact that we have no reason to believe *a priori* that tweets collected later in the month would display a different language pattern (for users with multiple records emerging from the spritzer).

## Conclusions

This paper set out to answer two research questions:

RQ1: Are there any demographic differences between users who do or do not enable local services on Twitter?

RQ2: Are there any demographic differences between users who do or do not geotag their tweets?

The answer to both of these questions is yes for all the demographic characteristics examined. There are clearly statistically significant differences based on demographic characteristics that effect the tendency to enable location services and geotag tweets, but the effect of the association is stronger for some characteristics than others. The size of the datasets used in this analysis mean that we can be confident that the differences we have found are not due to random chance, but in turn a large *n* will result in very small differences having very low *p*-values. It is therefore essential to consider the strength of association via the effect sizes or proportional differences for categorical data and the differences in means when comparing distributions where the differences are apparently small.

Whilst female tweeters are more likely than males to enable location services (by 0.8%) and males more likely to geotag their tweets than females (by 0.1%), the difference in proportions is quite small as are the associated effect sizes. Of those who enable location services, users with unisex names show the sharpest decrease in geotagging proportionally. The differences in age are also significant but small, with those who enable location services on average 0.55 years older than those who do not whilst those who used geotagging were 0.82 years older than those who did not. As both these differences are less than a year we may surmise that it is the long tail of older users who are causing the mean age to rise of a sample that is typically dominated by people under the age of 25 [17]. The differences between NS-SEC groups are of greater note in relation to those who enable location services, with a difference of 4.6% between the highest and lowest categories (semi-routine and routine respectively). However this gap shrinks to only 0.9% when considering rates of geotagging. As the class-based analysis could only be applied to users in the UK (using timezone as a proxy), these results cannot be generalised beyond the UK Twitter population.

The biggest differences in geolocation-based behaviour are related to language–both of the user interface and the tweet. Users who accessed the Russian Twitter interface and/or tweeted in Russian were the least likely to have geoservices enabled although these users were not in the lowest group for rate of geotagging, indicating perhaps that users wary of location services in this group tend to opt out at the global setting stage rather than enabling the setting and then choosing not to geotag. Turkish, Portuguese and Indonesian interface users are amongst the most likely both to enable the global setting and geotag tweets, with Portuguese and Indonesian language users also showing high proportions of users enabling geolocation and geotagging tweets.

Whether these differences are cultural or whether user interface language and/or tweet language can be considered a proxy for location are interesting questions. In the case of location proxy we may make some tentative observations about technological infrastructure and levels of smartphone use and it may be the case that decisions about behaviour on Twitter are primarily cultural for some groups but a function of technological necessities for others, or even a mix of both. Regardless of the cause, clear differences exist based on language that demonstrates inconsistent adoption of geoservices and geotagging. Further work on why this may be the case would be a fruitful future avenue of research.

To conclude, Graham et al. [23] were correct in their suspicion that the use of geoservices on Twitter is dependent on other demographic characteristics and whilst the behavioural differences related to gender and age may be small (if significant), economic and social class differences can be sizeable between particular groups and differences based on language are substantial. Those who enable the location setting and, perhaps more importantly, those who geotag their tweets are not representative of the wider Twitter population. For researchers, the impact of these findings will differ in magnitude depending on the topic being studied. For those using geotagged data from the 1% Twitter API the gender, age and class differences may be tolerable but careful consideration of the heterogeneity apparent in geoservice enablement and geotagging based on language (perhaps as a function of cultural and technological factors) is essential.
